# The role of endplate injury in intervertebral disc degeneration after vertebral augmentation in OVCF patients

**DOI:** 10.3389/fsurg.2022.1091717

**Published:** 2023-01-10

**Authors:** Yongchao Li, Qihang Su, Xiaofei Feng, Lijun Li, Jun Tan, Rongjun Ke

**Affiliations:** ^1^ Department of Orthopedics, Shanghai East Hospital, School of Medicine, Tongji University, Shanghai, China; ^2^ Department of Orthopedics, The Third Medical Center of Chinese PLA General Hospital, Beijing, China; ^3^ Department of Orthopedics, Tongji Hospital, School of Medicine, Tongji University, Shanghai, China; ^4^ Department of Orthopedics and Traumatology, Zhenjiang Hospital of Chinese Traditional and Western Medicine, Zhenjiang, China

**Keywords:** endplate injury, intervertebral disc degeneration, osteoporotic vertebral compression fractures, vertebral augmentation, magnetic resonance imaging

## Abstract

**Background:**

Whether vertebral augmentation can induce or aggravate the degeneration of adjacent intervertebral discs remains controversial. The purpose of this study is to explore the role of endplate injury in intervertebral disc degeneration after vertebral augmentation.

**Methods:**

The imaging data of patients with single-segment osteoporotic vertebral compression fractures (OVCFs) were retrospectively analyzed. The upper and lower discs of the fractured vertebrae were defined as cranial and caudal discs, and the discs adjacent to the cranial discs were defined as control discs. According to the integrity of the cranial and caudal endplates, they were divided into an injury group and a noninjury group. At follow-up, the increase in the modified Pfirrmann score on MRI compared with the baseline grade was defined as the occurrence of a degenerative disc change (DDC). The changes in the disc height and the number of DDC cases on MRI during the follow-up in each group were analyzed.

**Results:**

A total of 56 patients with OVCFs were included in this study, with an average follow-up time of 18.8 ± 14.1 months (3–62 months). In the cranial and caudal discs, the number of DDC cases in the endplate injury group was significantly higher than that in the noninjury group (*P* = 0.007 and *P* = 0.018). However, the number of DDC cases in the whole endplate injury group (including the cranial and caudal endplates) was significantly higher than that of the whole noninjury group (*P* = 0.000) and the control group (*P* = 0.000). The number of DDC cases in the whole noninjury group was not different from that of the control group (*P* = 0.192). At follow-up, the disc height of the cranial and caudal endplate injury group was significantly lower than the baseline (*P* = 0.000 and *P* = 0.001), but the disc height of the noninjury group was not significantly lower than the baseline (*P* = 0.074 and *P* = 0.082).

**Conclusion:**

Endplate injury is associated with adjacent intervertebral disc degeneration in OVCF patients after vertebral augmentation. Evaluation of endplate damage before vertebral enhancement in OVCF patients has an important reference value for predicting the outcome of adjacent intervertebral discs after surgery.

## Introduction

At present, whether vertebral augmentation can induce or aggravate the degeneration of adjacent intervertebral discs remains controversial. In several adult animal models (goats, sheep and dogs), the use of bone cement to block the endplate nutritional pathways for a certain period of time (months to 1.5 years) did not result in obvious adjacent disc degeneration (1–3). However, in immature animal models, the opposite result was obtained (4, 5).

In clinical studies, there are also inconsistent results, similar to the animal experiments. In 2012, Qian et al. (6) first clinically focused on the correlation between vertebral augmentation and intervertebral disc degeneration. They conducted a prospective study to compare the MRI data of OVCF patients with vertebral augmentation infused with polymethylmethacrylate (PMMA) bone cement (62 cases) and conservative treatment (35 cases). After a 2-year follow-up, they found that the incidence of upper intervertebral disc degeneration in the bone cement injection group (52.5%) was significantly higher than that in the conservative treatment group (29.0%) (*P* = 0.033). Subsequently, a retrospective imaging study conducted by Lu et al. (7) found that compared with conservatively treated patients, patients undergoing vertebral augmentation surgery had more severe degeneration of adjacent intervertebral discs (a decreased MRI signal). However, a long-term follow-up study (average 94.3 months) by König et al. (8) found that vertebral body cement enhancement had no significant effect on the degeneration of the intervertebral discs, and two-segment vertebral cement injection did not aggravate the degeneration of the sandwich intervertebral disc.

The nutritional support of the endplate to the intervertebral disc is contradictory to the structural support of the spine, which makes the endplate prone to damage (9, 10). After endplate injury, the bone marrow of the vertebral body contacts nucleus pulposus cells, which have immune privileges and can easily produce sustained inflammatory reactions during the process of injury repair (9). A series of mechanical tests (11), *in vitro* culture (12) and animal experiments (13–15) suggest that endplate injury may be a potential initiating factor of intervertebral disc degeneration. Therefore, in a study to determine whether injecting bone cement into fractured vertebrae can induce adjacent intervertebral disc degeneration, if the key factor of endplate injury is not controlled and analyzed, the conclusion may be unreliable. The purpose of our study was to evaluate the effect of endplate injury on adjacent intervertebral discs by retrospective imaging analysis and to explore the role of endplate injury in intervertebral disc degeneration after vertebral augmentation.

## Methods

### Study population

The clinical and imaging data of OVCF patients treated with vertebral augmentation from August 2013 to August 2020 were retrospectively analyzed. A total of 56 cases were included, including 20 males and 36 females, aged 68.2 ± 6.8 years (60–77 years), bone density of −2.72 ± 0.26, and average follow-up time of 18.8 ± 14.1 months (3–62 months). All patients were in the prone position, under local anesthesia, and injected *via* a unilateral pedicle approach, and the average amount of PMMA bone cement was 2.68 ml (1.5–4.5 ml).

Inclusion criteria: age ≥60 years old; preoperative MRI confirmed single vertebral fresh compression fractures of the lumbar spine (L1–L5), which showed low signal on T1 weighted images, high signal on fat suppression images and T2 weighted images (6), and the posterior wall of the vertebral body was intact without compression of the spinal cord or cauda equina; preoperative MRI showed that the modified Pfirrmann score of the adjacent intervertebral discs above and below the fractured vertebral body was ≤2 (16); the bone density T value was ≤−2.5 (17); vertebral augmentation was performed by the same group of doctors; and MRI data was available with postoperative follow-up ≥3 months.

Exclusion criteria: a previous history of lumbar surgery; leakage of bone cement into the intervertebral space during the vertebral augmentation; pathological fracture caused by tumor or infection; injury of adjacent intervertebral discs by the fractured vertebral body as observed on preoperative MRI, presenting as intervertebral disc edema or a focal high signal (18).

### Lumbar MRI imaging

A 1.5 T scanner (Achieva, Philips Medical Systems, Netherlands) was used for lumbar MRI imaging. Sagittal T1WI parameters: TR 450 ms, TE 17 ms. Sagittal T2WI parameters: TR 3500 ms, TE 80 ms. The SPAIR sequence (TR 2500 ms, TE 39 ms) was used to obtain fat suppression images (FSIs). FOV 160 × 160 mm, layer thickness 4 mm, layer spacing 3.6 mm. Matrix reconstruction 256 × 318.

### Imaging evaluation

**Figure 1:**
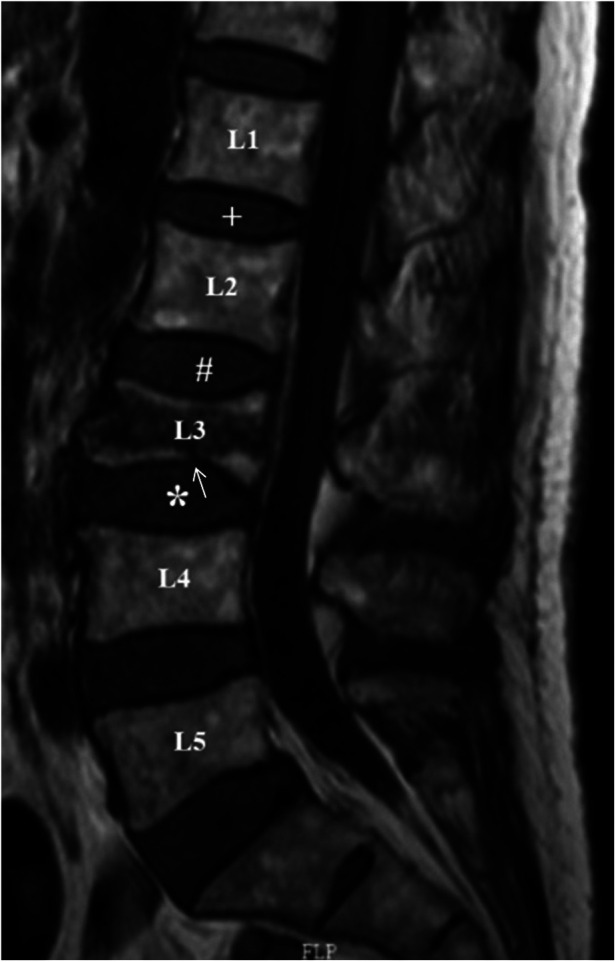
Definition of cranial, caudal and control discs. In the mid-sagittal view of MRI T1WI, L3 is the fractured vertebral body. L2/3 disc (#) and L3/4 disc (*) were defined as the cranial and caudal adjacent discs, respectively, and L1/2 disc (+) was defined as the internal control disc. The white arrow indicates caudal endplate injury of L3.

The upper and lower discs of the fractured vertebrae were defined as cranial discs and caudal discs, respectively, and the discs adjacent to the cranial discs were defined as control discs (Figure 1) (7). In addition, the upper and lower endplates of the fractured vertebrae were defined as the cranial endplate and caudal endplate, respectively.

The degree of disc degeneration was evaluated and recorded by the modified Pfirrmann scoring system (16). The improved modified Pfirrmann score on MRI was defined as degenerative disc changes (DDCs) compared with the preoperative MRI grade (excluding cases from level 1 to level 2). According to Lu et al.'s method (7), the heights of the cranial, caudal and control intervertebral discs were measured on mid-sagittal T1WI. Intervertebral disc height was defined as the average anterior, middle and posterior disc height. The kappa values of the interobserver and intraobserver methods were 0.89 and 0.78, respectively (7). On MRI T1WI and FSI, endplate injury was defined as continuous interruption or angulation of the endplates (Figure 1). According to the situation of endplate injury, they were divided into an injury group and a noninjury group.

### Statistical analysis

The data were analyzed by SPSS 23.0 (Chicago, United States). The counting data were expressed as percentages, and the measurement data were expressed as the mean ± standard deviation. Pearson's chi square test or continuous correction test was used to compare the incidence of cranial and caudal endplate injury and the number of DDCs among the cranial, caudal and control discs. At follow-up and baseline MRI, a paired t-test was used to compare the disc height changes between the cranial, caudal and control groups. The significance level was set at *P* < 0.05.

## Results

In this study, a total of 56 osteoporotic fractured vertebrae were included, for which the incidence of L1 vertebral fractures was the highest (41%). In 56 vertebral bodies, 55.4% of vertebral bodies had endplate injuries. Among them, 32.1% of the vertebral bodies had only cranial endplate injuries, 5.4% of the vertebral bodies had only caudal endplate injuries, and 17.9% of the vertebral bodies had cranial and caudal endplate injuries. The total incidence of cranial endplate injury was 50.0%, which was much higher than that of caudal endplate injury (23.3%) (*P* = 0.003).

**Figure 2:**
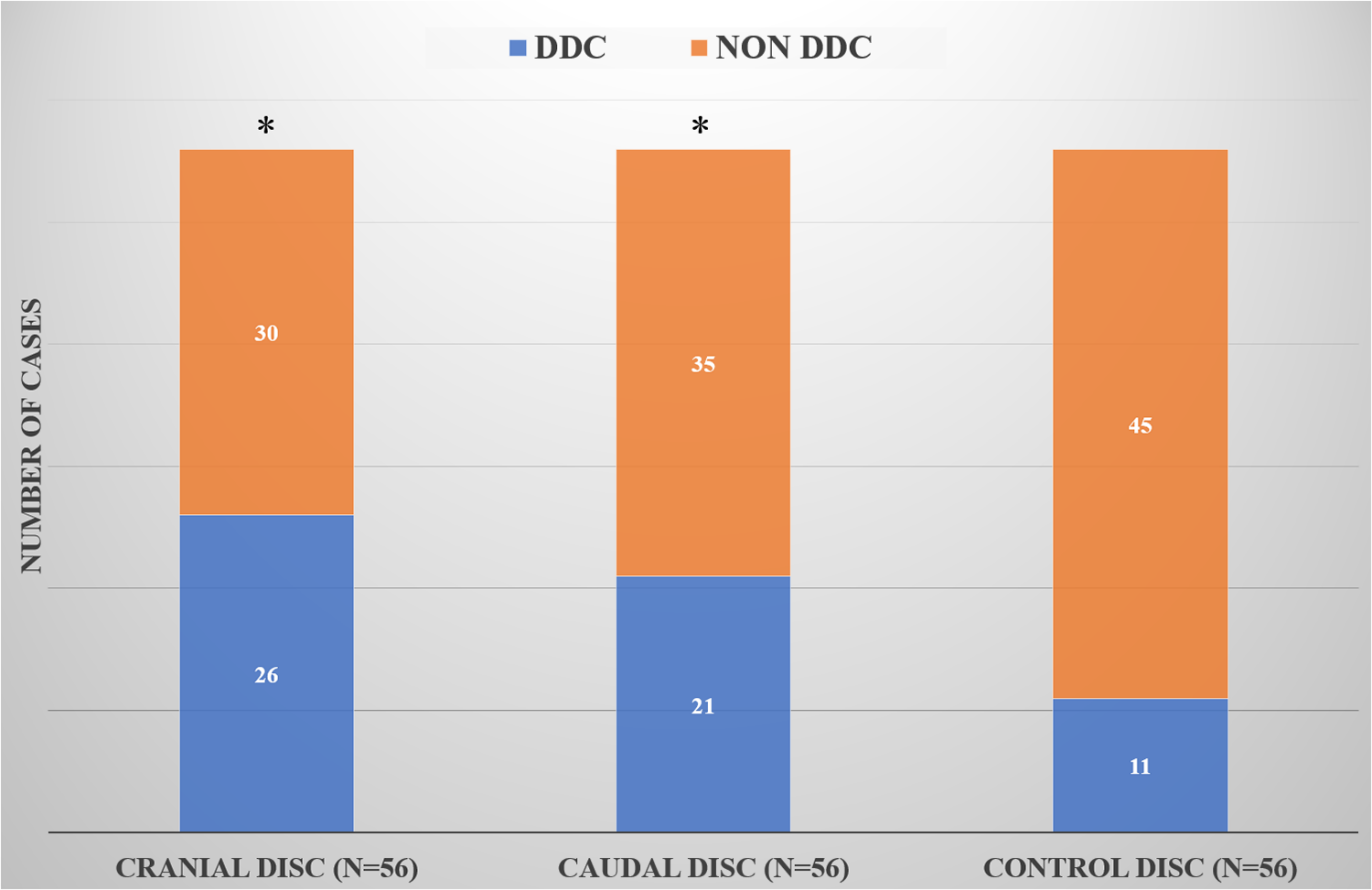
Comparison of DDC cases in the cranial, caudal and control groups. *Compared with the control group, there was a statistical difference.

At follow-up, the number of cranial and caudal DDC cases was significantly higher than that in the control group (*P* = 0.003 and *P* = 0.036), but there was no significant difference in the number of cranial and caudal DDC cases (*P* = 0.338) (Figure 2).

**Figure 3:**
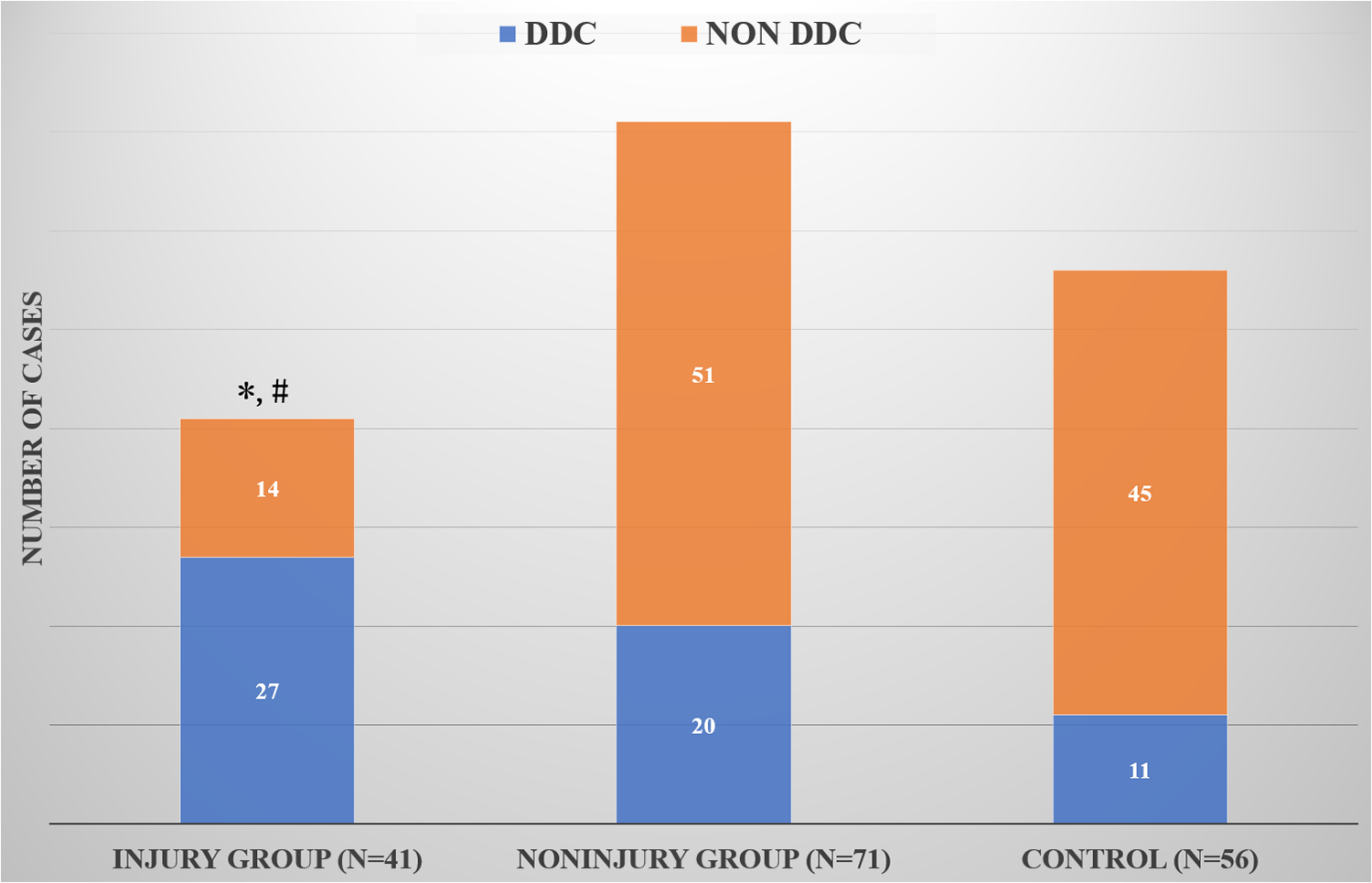
Comparison of DDC cases among the whole injury group, whole noninjury group and control group. *Compared with the control group, there was a significant difference; #Compared with the whole noninjury group, there was a significant difference.

In the cranial and caudal discs, the number of DDC cases in the endplate injury group was significantly higher than that in the noninjury group (*P* = 0.007 and *P* = 0.018) (Table 1). However, the number of DDC cases in the whole endplate injury group (including the cranial and caudal endplates) was significantly higher than that of the whole noninjury group (*P* = 0.000) and the control group (*P* = 0.000), but the number of DDC cases in the whole noninjury group was similar to that of the control group (*P* = 0.192) (Figure 3).

**Table 1:** The impact of cranial and caudal endplate injury on DDC (number of cases).

	Group	DDC	Non DDC	*P*
**Cranial disc**	Injury group (*n* = 28)	18	10	0.007
	Noninjury group (*n* = 28)	8	20	
**Caudal disc**	Injury group (*n* = 13)	9	4	0.018
	Noninjury group (*n* = 43)	12	31	

At follow-up, the height of the cranial and caudal discs was lower than that at baseline (*P* = 0.000 and *P* = 0.042), but there was no significant change in the disc height in the control group (*P* = 0.119). The intervertebral disc height in the cranial and caudal endplate injury groups was significantly lower than that at baseline (*P* = 0.000 and *P* = 0.001), but the intervertebral disc height in the noninjury group was not significantly lower than that at baseline (*P* = 0.074 and *P* = 0.082), as shown in Table 2.

**Table 2:** Changes in intervertebral disc height in the cranial, caudal and control groups.

Group	Intervertebral disc height		*P*
	Baseline	Follow-up	
**Cranial disc**	6.2 ± 1.7	5.7 ± 1.8	0.000
Injury group	6.2 ± 1.9	5.6 ± 1.9	0.000
Noninjury group	6.3 ± 1.6	6.0 ± 1.7	0.074
**Caudal disc**	7.4 ± 1.9	7.1 ± 2.1	0.042
Injury group	7.3 ± 1.9	6.9 ± 1.9	0.001
Noninjury group	7.5 ± 1.6	7.2 ± 1.7	0.082
**Control disc**	7.5 ± 1.3	7.3 ± 1.4	0.119

## Discussion

Regarding the complications after vertebral augmentation, scholars often focus on the impact of bone cement injection on adjacent vertebral bodies but rarely on adjacent intervertebral discs (19). Biomechanical studies have shown that bone cement can increase the mechanical stiffness of the fractured vertebral body, thus changing the load transfer to the adjacent intervertebral disc and vertebral body, which may lead to a fracture of the adjacent vertebral body (20). Some studies (20, 21) have found that vertebral augmentation can significantly increase the incidence of adjacent vertebral fractures, but other systematic reviews and meta-analyses have found no evidence that vertebral augmentation can increase the incidence of adjacent vertebral fractures (19, 22, 23). The occurrence of adjacent vertebral fractures is only based on anecdotal reports (19, 22, 23).

Similarly, the effect of vertebral augmentation on adjacent discs is controversial. In several adult animal models (goats, sheep and dogs), the use of bone cement to block the endplate nutritional pathways for a certain period of time (months to 1.5 years) did not find obvious disc degeneration (1–3). However, in immature pig and rabbit animal models, the opposite conclusion was obtained (4, 5). Clinical studies have shown similar inconsistent results. Both prospective studies by Qian et al. (6) and retrospective imaging studies by Lu et al. (7) reported that the injection of bone cement into the fractured vertebral body could increase the incidence of adjacent intervertebral disc degeneration. However, a long-term follow-up study (average 94.3 months) by König et al. (8) found that PKP had no significant effect on the adjacent intervertebral discs, and the injection of bone cement into both adjacent segments did not cause or aggravate the degeneration of the sandwich intervertebral discs. However, in the animal experiments, bone cement was injected into a vertebral body with normal bone, ignoring the fact that the vertebral body mainly used for the treatment of OVCFs is osteoporotic and fractured.

In addition to the bias caused by these design defects, the above clinical studies ignored or did not explore the influence of endplate injury as a key variable. In our study, we divided OVCF patients into an endplate injury group and a noninjury group according to the integrity of the endplate on preoperative MRI. The results showed that the number of DDC cases in the endplate injury group was significantly higher than that in the noninjury group (*P* = 0.007 and *P* = 0.018). Additionally, the number of DDC cases in the whole endplate injury group (including the cranial and caudal endplates) was significantly higher than that in the whole noninjury group (*P* = 0.000) and the control group (*P* = 0.000), but there was no significant difference between the whole noninjury group and the control group (*P* = 0.192). It has been suggested that endplate injury is a risk factor for accelerated degeneration of the intervertebral disc adjacent to the OVCF vertebral body. However, if the endplate injury variable is excluded, the effect of bone cement injection on the cranial and caudal intervertebral discs is not obvious.

In addition, our study also found that the cranial and caudal disc heights were lower than the baseline at follow-up (*P* = 0.000 and *P* = 0.042), but there was no significant change in the disc height in the control group (*P* = 0.119). The results of this study are similar to those of Lu et al. (7). However, we also found that the disc height in the cranial and caudal endplate injury groups was significantly lower than that at baseline (*P* = 0.000 and *P* = 0.001), but there was no significant decrease in the noninjury group (*P* = 0.074 and *P* = 0.082). These results indicate that cranial and caudal disc endplate injuries have significant effects on adjacent disc height and further confirmed that endplate injury might play an important role in adjacent disc degeneration after OVCF vertebral augmentation.

The pathogenesis of disc degeneration is still unclear, but the lack of a nutrition supply may be one of the important factors leading to disc degeneration (4, 24). The main nutritional pathway of intervertebral discs comes from the diffusion of adjacent endplates (24, 25). Theoretically, the filling of bone cement into the fractured vertebrae, especially bone cement adjacent to the endplate, may affect the nutrient blood vessels, resulting in the degeneration of adjacent intervertebral discs after vertebral augmentation (5). However, injection of bone cement into the vertebrae of mature animals did not cause adjacent disc degeneration (1–3). Kang et al. (4) suggested that these negative results may be attributed to the remaining endplate penetration after bone cement injection, which can continue to provide sufficient nutrition for the intervertebral disc. Additionally, Krebs et al. (3) found that the formation of new bone around bone cement indicated that there was still a nutrient supply in the bone cement space. Previously, Ibrahim et al. (26) found that compared with mature animals, immature animals have significantly greater diffusion through the endplates, and greater nutritional requirements may make these discs more susceptible to changes in nutritional supply (4). Therefore, in immature animal models, studies have found that vertebral injection of bone cement can cause adjacent intervertebral disc degeneration (4, 5). However, vertebral augmentation is mainly used in the treatment of elderly patients with OVCFs.

We found that the number of DDCs in the noninjured endplate group after the injection of bone cement was not different from that in the control group, indicating that the collateral circulation without bone cement damage in the vertebral body is sufficient to provide nutrition for the elderly intervertebral disc. Therefore, for OVCF patients with intact endplates, the injection of bone cement into the vertebral body may not induce degeneration of adjacent intervertebral discs.

The nutritional support of the endplate to the intervertebral disc is contradictory to the structural support of the spine, which makes the endplate prone to injury (9). Our study found that the incidence of endplate injury was 55.4%. The incidence of endplate injury in our study was lower than the 61% reported by Fujiwara et al. (10), which may be related to the different inclusion criteria. After endplate injury, the bone marrow of the vertebral body contacts nucleus pulposus cells, which have immune privileges and easily produce sustained inflammatory reactions during the process of injury repair (9). A series of mechanical tests (11), *in vitro* culture (12) and animal experiments (13–15) suggest that endplate injury may be a potential initiating factor of intervertebral disc degeneration. Therefore, we believe that OVCF patients with endplate injury is the most important reason for degeneration of the adjacent intervertebral disc after vertebral augmentation rather than the injected bone cement. This may explain the inconsistent conclusions in previous clinical studies on the effects of vertebral augmentation on adjacent intervertebral discs (6–8).

This study has some limitations. First, because of the small sample size, it is impossible to compare each fractured vertebral segment with others. Second, strict inclusion and exclusion criteria were established in this study, which may lead to sampling bias. Third, the average follow-up time was still short, which is not sufficient to fully explain the effect of bone cement injection into the fractured vertebral body on the adjacent intervertebral disc. Fourth, limited by retrospective imaging research, it is difficult to accurately identify microinjuries to the endplate or intervertebral disc by imaging, and further basic research is needed for verification. Fifth, the obvious limitation of our study is that the clinical data were not included, and the clinical relevance of the research results still needs to be further verified. Sixth, although the modified Pfirrmann scoring system has good assessment consistency, further research is needed to verify the consistency of this conclusion by using quantitative indicators such as the intervertebral disc index (27), T2 mapping (28) or apparent diffusion coefficient (29).

## Conclusion

Endplate injury is associated with adjacent intervertebral disc degeneration after vertebral enhancement in OVCF patients. Evaluation of endplate damage before vertebral enhancement in OVCF patients has an important reference value for predicting the outcome of adjacent intervertebral discs after surgery.

## Data Availability

The original contributions presented in the study are included in the article/Supplementary Material, further inquiries can be directed to the corresponding author/s.
